# Experimental High-Resolution Observation of the Truncated Double-Icosahedron Structure: A Stable Twinned Shell in Alloyed Au–Ag Core@Shell Nanoparticles

**DOI:** 10.1021/acs.nanolett.3c04435

**Published:** 2024-04-01

**Authors:** Rubén Mendoza-Cruz, Juan Pedro Palomares-Báez, Stephan Mario López-López, Juan Martín Montejano-Carrizales, José Luis Rodríguez López, Miguel José Yacamán, Lourdes Bazán-Díaz

**Affiliations:** † Instituto de Investigaciones en Materiales, Universidad Nacional Autónoma de México, Circuito Exterior, Ciudad Universitaria, Ciudad de México, Mexico 04510; ‡ Facultad de Ciencias Químicas, Universidad Autónoma de Chihuahua, Circuito Universitario s/n, Campus II, Chihuahua, Mexico 31125; § Posgrado en Ciencia e Ingeniería de Materiales, Universidad Nacional Autónoma de México, Circuito Exterior, Ciudad Universitaria, Ciudad de México, Mexico 04510; ∥ Instituto de Física, Universidad Autónoma de San Luis Potosí, San Luis Potosí, Mexico 78290; ⊥ Advanced Materials Department, Instituto Potosino de Investigación Científica y Tecnológica, A.C., San Luis Potosí, Mexico 78216; # Department of Applied Physics and Materials Science and MIRA, Northern Arizona University, Flagstaff, Arizona 86011, United States

**Keywords:** Au−Ag nanoparticles, decahedral nanoparticles, icosahedral nanoparticles, double icosahedron, multiply twinned nanoparticles, nanoalloys

## Abstract

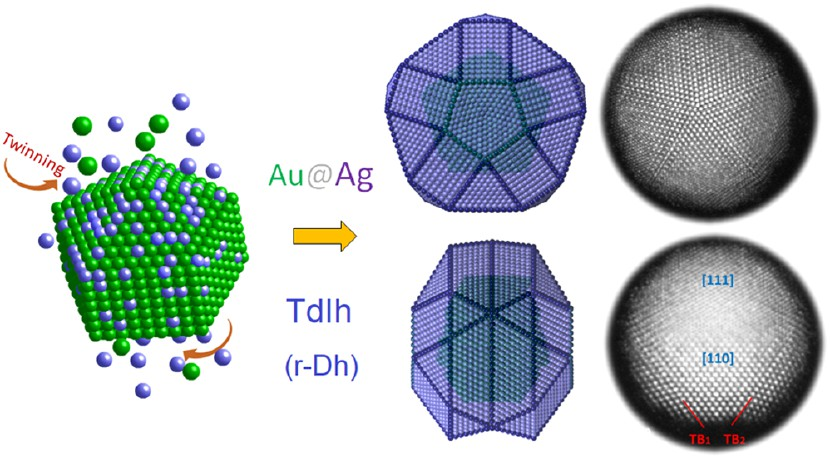

Given the binary nature of nanoalloy systems, their properties are dependent on their size, shape, structure, composition, and chemical ordering. When energy and entropic factors for shapes and structure variations are considered in nanoparticle growth, the spectra of shapes become so vast that even metastable arrangements have been reported under ambient conditions. Experimental and theoretical variations of multiply twinned particles have been observed, from the Ino and Marks decahedra to polyicosahedra and polydecahedra with comparable energetic stability among them. Herein, we report the experimental production of a stable doubly truncated double-icosahedron structure (TdIh) in Au–Ag nanoparticles, in which a twinned Ag-rich alloyed shell is reconstructed on a Au–Ag alloyed Ino-decahedral core. The structure, chemical composition, and growth pathway are proposed on the basis of high-angle annular dark-field scanning transmission electron microscopy analysis and excess energy calculations, while its structural stability is estimated by large-scale atomic molecular dynamics simulations. This novel nanostructure differs from other structures previously reported.

Bimetallic nanoparticles (NPs) have been used in essential areas in the past few years due to their unique properties. Because of the binary nature of nanoalloys, these properties can be tuned by not only the size but also the structure, composition, and chemical ordering.^1,2^ Au–Ag nanoalloys have important applications in optoelectronics, plasmonics, molecular sensing, catalysis, and biomedical areas.^3−9^ Au–Ag NPs present silver surface segregation when thermodynamic equilibrium is reached owing to their lower free surface energy,^10−12^ enabling tuning of their optical response.

At the nanoscale, the bulk atomic arrangement changes, and new structures or atomic ordering emerges; furthermore, structural defects modify the morphology, structure, and properties. Noble-metal NPs crystallize with the bulk-stable face-centered cubic (FCC) structure with a variety of shapes through facet truncations (dodecahedra, octahedra, cubes, etc.) or into multiply twinned particles (MTPs, decahedra, or icosahedra) and some habit variations. The possibilities of shape variations are vast, even if they are transitional metastable structures during the formation or transformation into more stable configurations.^13−16^ Icosahedra (Ih) and decahedra (Dh) are common structures,^13,17^ but their formation depends on the synthesis methodology, the origin of structural variations that compete with stability.

Among the surface reconstruction phenomena,^18^ truncation is an efficient way to optimize the energetic stability of NPs. This leads to the appearance of new facets modifying the total surface energy^16^ or anisotropic growth.^19,20^ Experimental and theoretical observations of MTP variations have been reported, such as the Ino-decahedron (I-Dh),^21^ Marks decahedron (m-Dh),^22^ Decmon families (dm-Dh),^16^ Mackay icosahedron (Ih),^23,24^ Chui icosahedron (c-Ih),^25,26^ truncated icosahedra (TIh),^18,27^ and others beyond a single MTP such as the bidecahedron (bi-Dh),^28^ double icosahedron (dIh),^29,30^ or further polyicosahedral and polydecahedral structures.^31−35^

The formation of twinned units by surface reconstruction in the initial growth stages decreases the total energy because Dh or Ih is more stable than a single tetrahedron.^36^ Then, overgrowth would generate bi- or tri-tetrahedra instead of following the tetrahedral shape, subsequently forming MTP structures. Recent studies demonstrated that the growth pathways for Dh and Ih formation depend on how initial tetrahedra form and evolve.^36−39^ These initial tetrahedra can stabilize new twin boundaries (TB) overgrowing into Dh or, subsequently, Ih.^39^ Moreover, the development of faulted layers during crystal growth can be induced by a second metal in nanoalloys,^40−42^ inducing surface reconstruction and decreasing the free surface energy to stabilize uncommon structures.^18,35,43,44^ In this way, bimetallic systems present unusual MTP structures shaped from structural defects and composition inhomogeneities, which has repercussions in the free surface energy minimization in the small size regime.

Herein, we present the unreported experimental observation of a truncated MTP structure in Au–Ag NPs. Aberration-corrected high-angle annular dark-field (HAADF) imaging enabled the determination of the structural features of the nanoalloy.^45−49^ On the basis of the experimental images and supported by theoretical calculations, we proposed that the Au–Ag NPs adopted an alloyed core@shell doubly truncated double-icosahedron (T-dIh) structure so that a twinned Ag-rich shell is formed on a Au–Ag decahedral core.

The Au–Ag NPs were produced by a wet-chemistry method adapting the methodology described elsewhere^11^ (for details, see the Supporting Information). Transmission electron microscopy (TEM) images in Figure 1a confirm the production of MTP with an average size of 10.1 ± 2.6 nm. The size histogram and selected-area electron diffraction (SAED) patterns are shown in Figure S1. The SAED pattern resembled the structure of silver/gold alloys. Because of their very similar cell parameters (*a*_Au_ = 4.078 Å, and *a*_Ag_ = 4.086 Å), SAED or conventional TEM on Au–Ag NPs is difficult, hindering the atomic ordering. HAADF imaging allowed the visualization of a core–shell-type configuration, as shown in Figure 1b. The core diameter was 6.8 ± 0.8 nm with an average shell thickness of 1.6 ± 0.2 nm. The particles crystallize into MTP structures, which are expected to form via OLA-based synthesis.^50^

**Figure 1:**
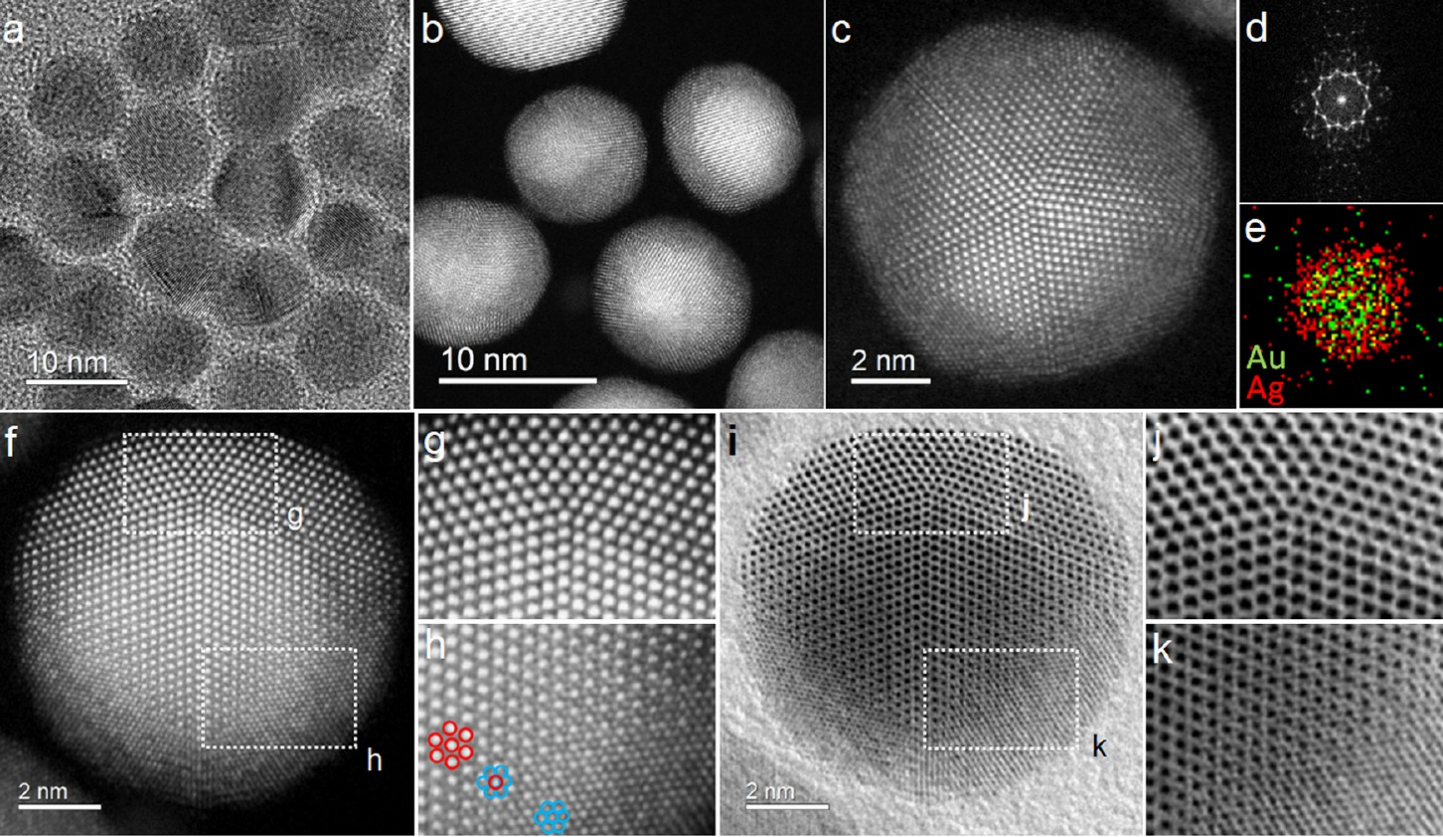
(a) TEM image of the synthesized NPs. (b) HAADF image in which the core@shell configuration can be seen. (c and d) High-magnification HAADF image of a single NP and its corresponding FFT highlighting its 5-fold symmetry. (e) EDS mapping showing the Au–Ag NP composition with the preferential presence of Ag at the surface. (f–h) Atomic-resolution HAADF and (i–k) BF images of a single NP viewed along a secondary 5-fold axis from which the atomic arrangement is clearly resolved. (g, h, j, and k) Magnified images of the areas shown in panels f and i, respectively. In panel h, the circles highlight the hexagonal low-contrast pattern (blue circles) surrounded by a high-contrast hexagonal arrangement (red circles).

HAADF imaging on a single MTP along the 5-fold symmetry axis is shown in Figure 1c, together with its fast Fourier transform (FFT) (Figure 1d). The chemical composition was confirmed by energy dispersive X-ray spectroscopy (EDS) mapping, EDS line scans, and image analyses (Figure 1e and Figure S2). The produced NPs do not correspond to a complete Au–Ag alloy or pure core–shell configuration; instead, they consisted of an alloyed Au–Ag core surrounded by an alloyed Ag-rich shell. The average chemical composition was Au_24_Ag_76_, in good agreement with the initial precursor’s molar concentrations, while the core and shell compositions were estimated to be close to Au_50_Ag_50_ and Au_20_Ag_80_, respectively. Although Ag segregation has been recently challenged for MTP structures,^51^ our experimental conditions greatly differ from gas-phase methodologies, revealing the segregation of Ag to the surface. This is in agreement with the theoretical model applied by Guisbiers et al.^11^ in which the driving force for surface segregation in the first place is due to the null immiscibility of the alloy elements and in the second to thermodynamics, i.e., by minimization of the NP free energy. The second rule of the model applied to the Electrum Au–Ag alloy.

Interestingly, although the structural features are similar to those of Dh-NPs, atomic-resolution images revealed a different contrast (Figure 1f–k and Figure S3). We noted that (1) the atomic arrangement seems to change from the core to the surface, differing from the pure [110] projection of a regular Dh, (2) the projected contrast at the TB locations differs from that of a regular Dh, (3) the contrast varies from one individual atomic column to another (Figure 1h), and (4) the projected column width increases significantly from the surface to the core, being more evident for smaller particles. In response, we carefully analyzed the structural features and proposed structural models.

In HAADF imaging, the positions of atomic columns are directly determined.^52^ The lattice distances from monocrystalline domains were measured (Figure 2a–d and Figures S4 and S5). At the particle core, the distances (angle) of 0.23 and 0.19 nm (53°) matched the (11̅1) and (002) planes, respectively, of the Au/Ag structure, viewed along the [110] direction. However, near the surface, {111} reflections in the FFT vanished, preserving and intensifying the {113} and {220} reflections that match the [114] orientation. Between the core and the edges, the contrast varies from one individual atomic column to another (second observation described above); i.e., each brighter column is surrounded by six low-contrast columns, indicated by circles in Figure 1h.

**Figure 2:**
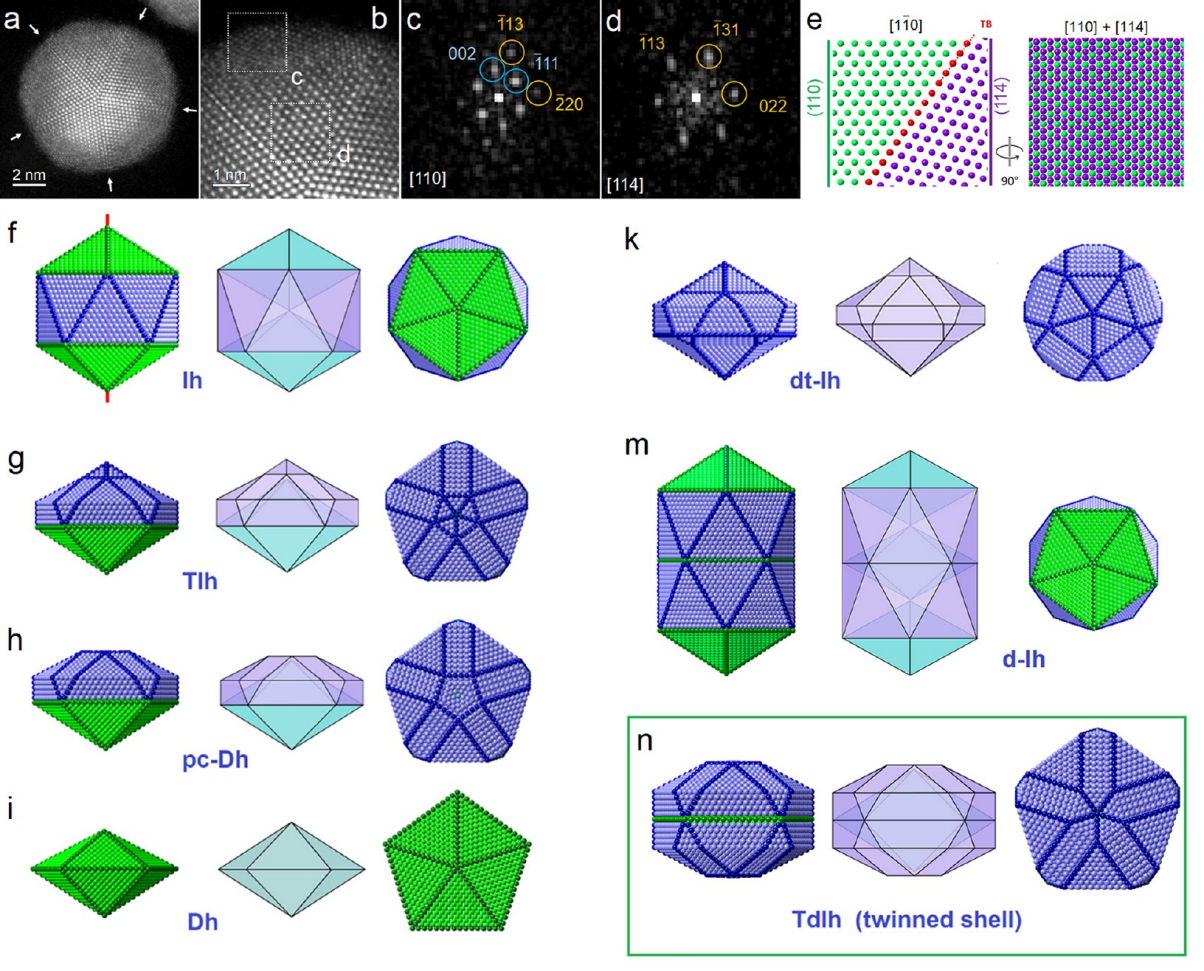
(a and b) High-magnification STEM-HAADF images showing the structural features at the twin boundaries and NP’s surface and (c and d) corresponding FFT analysis from the central and edge areas, respectively, shown in panel b. (e) Schematic of the atomic arrangement of a ∑3 {111} twin boundary viewed from the [11̅0] parent direction and rotated by 90° at the [110] parent direction in which both crystals overlap to form a [110] + [114] pattern. (f–i, k, m, and n) Shape variations of MTPs due to particle truncation. Geometrical deconstructions can be followed. From Ih to Dh pathway 1 (f–g–h–i), from Ih to dt-Ih pathway 2 (f–g–k), and from dIh to TdIh, pathway 3 (m–n).

The latter observations indicate that the projection corresponds to a superposition of two crystals oriented along the [110] and [114] directions sharing common planes. This superposition is observed, even at the core location. The [110] and [114] projections are related by a {111}-twin plane that is visible if the crystal is viewed parallel to it (Figure 2e). However, if the twinned crystal is viewed from a direction normal to the (110) planes, a superposition between the parent and the 60°-rotated twinned crystal is observed. The generated contrast along the superimposed direction can be difficult to interpret from conventional HRTEM images, as one can see in HRTEM simulations in Figure S6. This complication is surpassed in Cs-corrected HAADF imaging, where the positions of individual atomic columns are effectively resolved.^49^

The presence of TBs parallel to {111} facets of the core corresponds to a reconstructed Dh or truncated MTP, such as a TIh, in which the top layers of an Ih are removed. The regular Dh and Ih are closely related. An Ih can be described as the union of two regular Dh’s along their 5-fold axis, sharing a vertex atom but rotated 36° from each other.^27^ The remaining space is filled by 10 central tetrahedra sharing twinned planes. The Ih-to-Dh transition (or the inverse order in Dh reconstruction) can be seen as a continuous geometrical truncation of the Ih from its top layers, resulting in a regular Dh structure at the bottom, or, inversely, the overgrowth of twinned tetrahedra up to obtaining a complete Ih, generating intermediate uncommon but thermodynamically stable morphologies.^16^

Geometrical deconstructions from a regular Ih structure to different structures by truncation are sketched in panels f–i, k, m, and n of Figure 2. The two Dh forming the Ih along a main 5-fold axis are colored green, while the central tetrahedra are colored purple. It should be emphasized that these geometrical deconstructions are presented only to visualize the structure of the truncated MTPs, not the growth mechanisms. Atom-ball models of each structure along a main 5-fold axis are displayed to highlight their symmetry and shared features. Three geometrical pathways are presented. The first, denoted as f–g–h–i, starts from the Ih, and removal of the top layers gives rise to a single TIh with a characteristic small Dh tip formed by the remaining portions of the top Dh (Figure 2g). The appearance of long {100} facets is observed. This structure is known as the Montejano decahedron (Decmon).^16^ Now, if the apical Dh tip is removed, then a partially covered decahedron (pc-Dh) is formed. This structure can be viewed as a TIh with a concavity at the top (Figure 2h).^38^ Total removal of the remaining central tetrahedra results in the regular Dh (Figure 2i). A second geometrical pathway is denoted as f–g–k, in which the Ih top and bottom layers are removed generating a doubly truncated Ih (dt-Ih) (Figure 2k). It can be noted that truncation modifies the external shape and reveals the features across TB.

A particular feature in Ih structures is the central pattern along the 5-fold axis produced by the overlapping of the Dh tip and the lower Dh, rotated 36° from each other. This feature is preserved in TIh and dt-Ih, but it is reduced if the Dh tip is removed. For this reason, HAADF-STEM images were simulated for each structure along the 5-fold axis and are shown in Figure S7. The experimental data showed a central feature more similar to a Dh or a pc-Dh structure, as one can see in panels f and i of Figure 1. Therefore, another model is proposed (pathway m–n). It consists of the same f–g–h pathway but applied to a dIh that shares a central Dh, truncating the top and bottom parts, including the two apical Dh’s (or, inversely, the reconstruction of a central Dh) (Figure 2m,n). Partial tetrahedra formed on all of the {111} facets of a Dh core by twinning. Hence, this model can be seen as a doubly truncated double icosahedron (TdIh) in which partial tetrahedra on the top and bottom facets of an alloyed Dh formed a twinned Ag-rich shell.

Images of particles at random orientations confirmed the TdIh structure. The particle in Figure 3a imaged near the main 5-fold symmetry shows {100} contrast indicating {100}-truncated facets. More importantly, lateral views in panels b and c of Figure 3 reveal the twinned layer on the top and bottom sides of an I-Dh core. In Figure 3b, an ∼162° angle is formed by the {111} and {200} planes of the parent (core) and shell (twinned), respectively, while the lateral view in Figure 3c shows twinned atomic columns and overlapping areas. Rotated magnified areas around the indicated TB_1_ and TB_2_ are presented in Figure 3d, providing a clear observation of the ABCBA... stacking on both sides of the NP core and confirming the TdIh structure. Panels e–h of Figure 3 and Figure S8 correspond to focal series of a single NP. When passing from over to under focus, the truncation along the TBs is observed. The complete series is presented in Movie S1.

**Figure 3:**
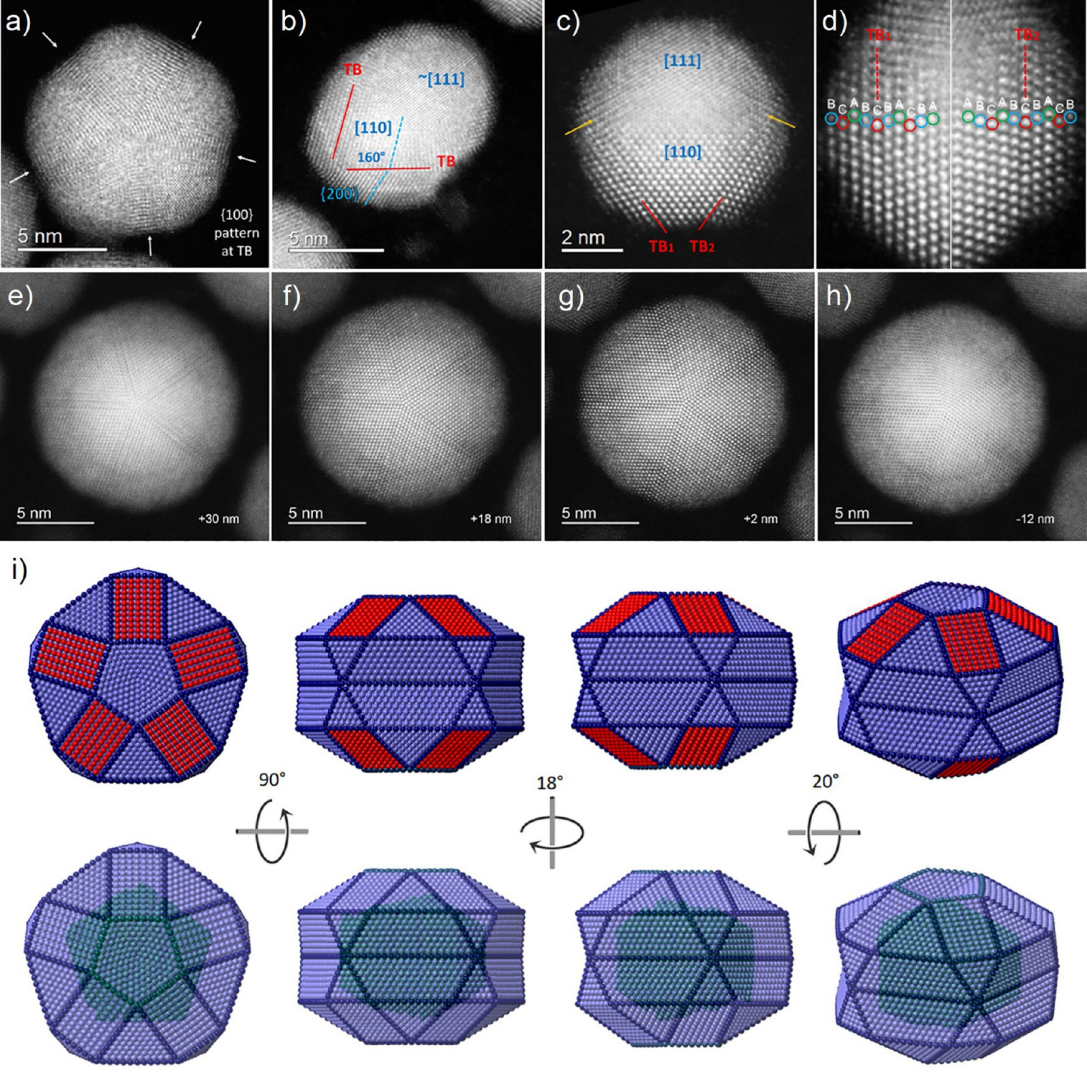
(a) High-magnification HAADF image of single Au–Ag NPs oriented near the main 5-fold axis. The arrows indicate the {100} contrast at the TBs that originate from truncation. (b and c) Lateral views of particles in which a doubly twinned layer is observed. The TB’s are indicated with a red line, and overlapping areas with a yellow arrow. (d) Rotated magnified areas around TB_1_ and TB_2_ shown in panel c, where the ABCBA... stacking sequence confirmed the presence of the twinned shell. It is noted that the shell consists of four to six twinned planes. (e–h) Focal series on a single TdIh. (i) Atom-ball model of the TdIh along the main 5-fold axis, lateral views, and a 20° tilt to show the absence of the Dh tip. The {100} facets are colored red (top row), and a transparent shell is presented to show the Au core (green, bottom row).

The experimental images doubtlessly confirmed the presence of a twin shell on an I-Dh core, generating a particular overlapped pattern not observed in other truncated structures. An atom-ball model of the TdIh at different projections is presented in Figure 3i, in which the concavity produced by the apical Dh subtraction resembles the experimental images.

HAADF simulations were performed considering a TdIh with an alloyed I-Dh Au_50_Ag_50_ core and Au_20_Ag_80_ shell. The simulated image in Figure 4a corresponds to the TdIh oriented along the main 5-fold axis, which is compared with the experimental image. Simulations along the lateral view and secondary 5-fold axis are also shown (Figure 4b). The high-contrast [110] pattern highlighted by the red hexagon in Figure 4a corresponds to the core projection, while the internal [114] pattern corresponds to the shell. These images correctly resembled the experimental ones, matching the atomic arrangement and the contrast variations across atomic columns, addressing the experimental observations: (1) the overlapped [110] + [114] projections, (2) the {100} features along the TB, (3) the atomic arrangement and compositional variations, and (4) the increasing column width from the edge to the NP center. Intensity variations across all of the atomic columns are produced by the mixed nature of the alloy, with some columns richer in one metal than others (Figure 4c). These contrast variations are similar in the simulated image (Figure 4d). To characterize the NP’s structure from different angles, tilting series were acquired (Figure S9). The models and simulated images had excellent concordance with the experimental structure.

**Figure 4:**
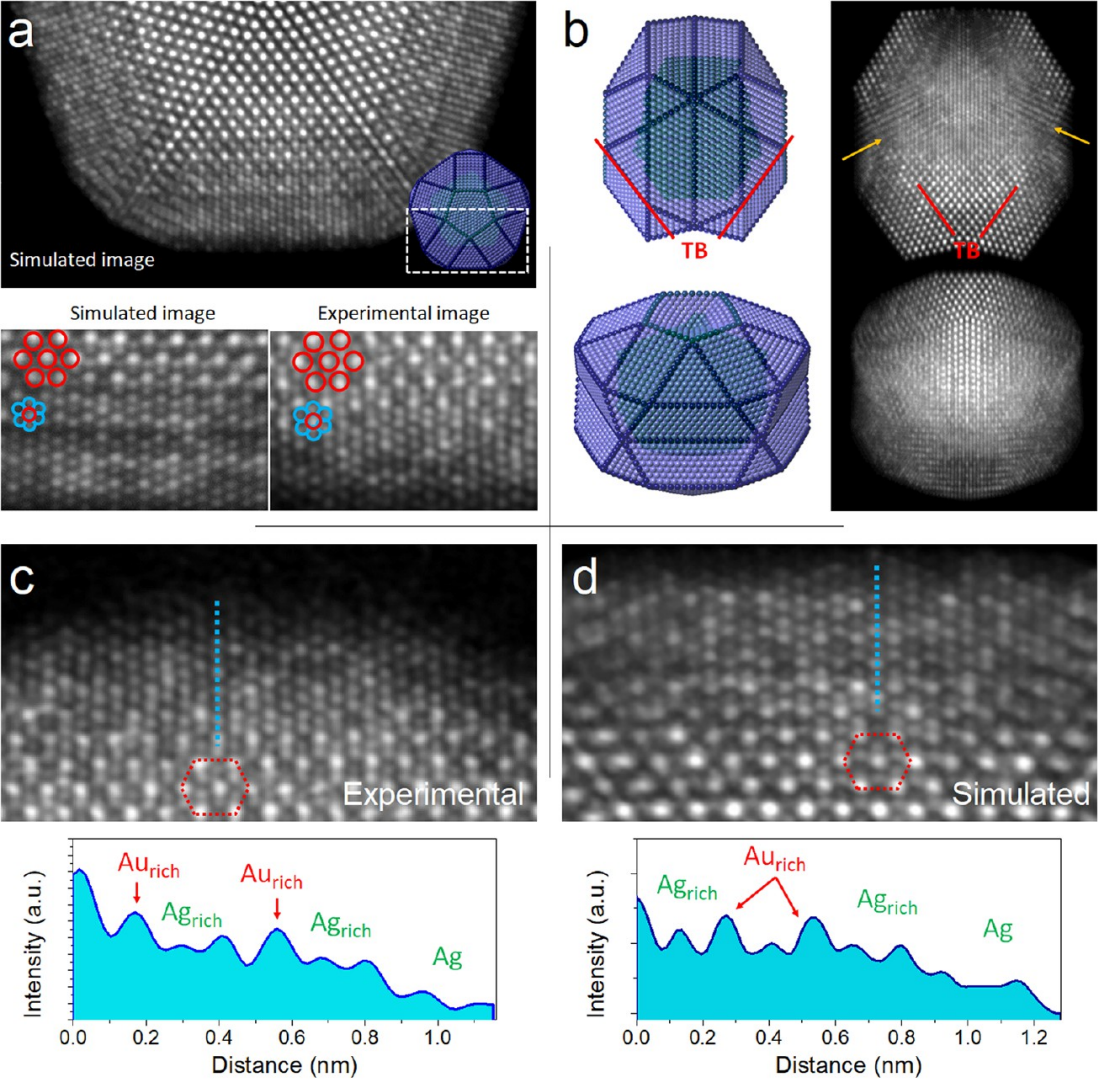
HAADF simulations of an Au–Ag TdIh oriented along (a) the main 5-fold axis (half of the TdIh is shown for the sake of clarity) and (b) lateral and secondary 5-fold axis at 60°. A comparison between the simulated and experimental images of a single domain is shown in panel a, highlighting the contrast variation across atomic columns. The high-contrast [110] pattern highlighted by the red hexagon corresponds to the core projection, and the internal [114] pattern to the shell projection. (c and d) Experimental and simulated images of a TdIh, respectively, and intensity profiles across atomic columns on the shell area. Intensity variations are produced by the mixed nature of the alloyed particle, with some columns richer in one metal than other, ending with Ag at the outer layers.

To understand the formation mechanism, the growth evolution was followed by taking aliquots at early stages of the reaction (Figures S10 and S11). At 5 min, a small I-Dh formed with an average composition of Au_71_Ag_29_, obeying the difference in reduction potentials of the precursors. Then, the silver concentration increased with time, inducing reconstruction after reaction for 6 min. After 15 min, the TdIh structure is visible, with so far small shells. At 90 min, the particles showed the TdIh structure with an overall Ag-rich composition (Au_27_Ag_73_). The method yielded >75% of TdIh, followed by single-crystal and Ih structures (Figure S12). At a higher temperature (150 °C), single-crystal NPs were favored, yielding less MTP. This behavior can be understood by the change in reduction kinetics with an increase in the reduction rate, diminishing MTP production,^53^ which is in agreement with experimental observations on the formation of MTP at low temperatures.^54−56^ In the absence of OAc, Dh was the preferential structure. The OLA/OAc mixture enhances the growth rate for anisotropic structures.^56−58^

To support the growth mechanism from an alloyed I-Dh to the TdIh structure, we calculated the excess energy (*E*_exc_) of growth pathways.^59^Figure 5a shows that starting from a pure Au core, *E*_exc_ is zero before the addition of atomic layers forming a pure Ag shell. A jump occurred when the first Ag layer was added (reconstruction of the I-Dh faces generating twinning, a necessary step for TdIh construction), favoring NP growth. However, subsequent addition of Ag layers increases *E*_exc_, making this pathway less favorable. In contrast, when the process starts from an alloyed I-Dh with the subsequent addition of Au–Ag alloyed layers, the growth pathway is always favored (compared with the pure Au and Ag particles). The same jump is observed when reconstruction occurs, attributed to the increase in Ag concentration. Moreover, the experimental Au–Ag composition is almost optimal to favor this pathway, as shown in Figure 5b, where *E*_exc_ increases for other Au–Ag proportions (overall composition of Au_24_Ag_76_) disfavoring TdIh formation. The pathway is shown in Movie S2.

**Figure 5:**
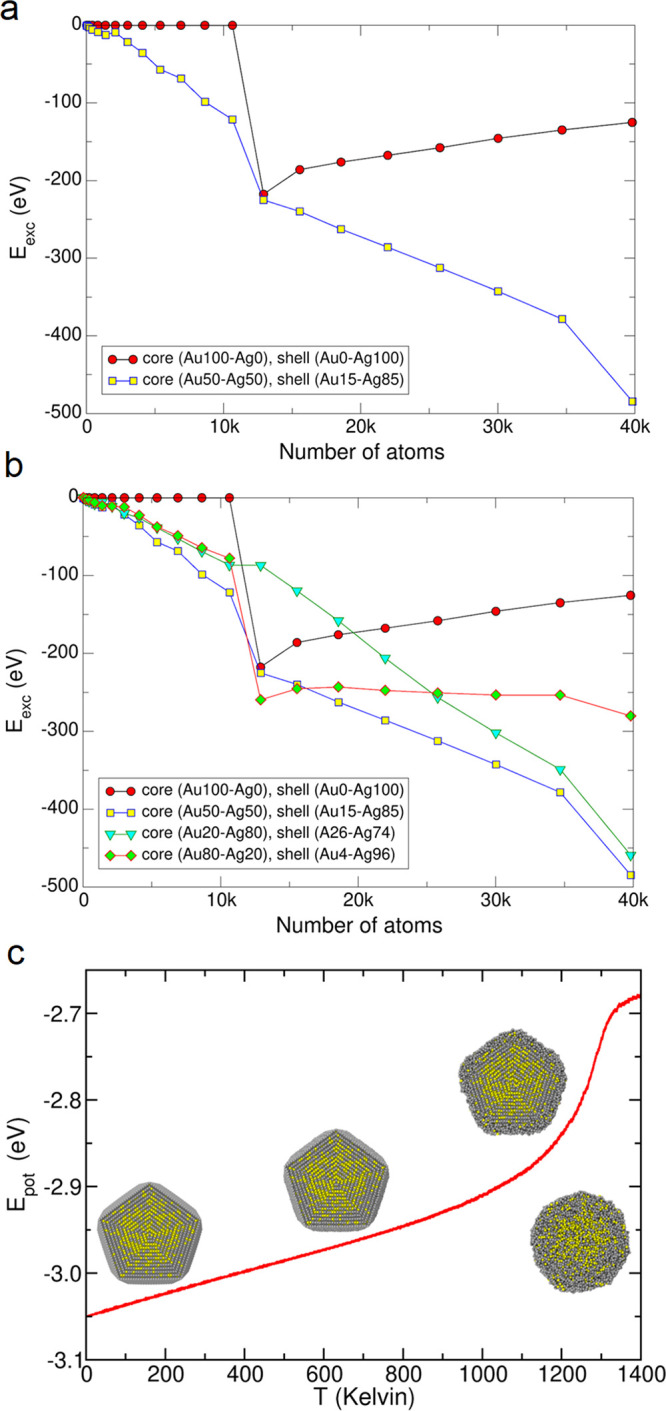
(a) Evolution of the excess energy, *E*_exc_, as a function of TdIh size. Two growth pathways are compared: the growth of an Au core on which a pure Ag shell is subsequently formed and the first growth of an alloyed Au_55_Ag_45_ core on which Au and Ag atoms deposit forming an alloyed shell with a Au_15_Ag_85_ composition. (b) Pathways for alloyed core and shells with different Au–Ag proportions. (c) Energy stability for the modeled alloyed Au–Ag core–shell TdIh, i.e., potential energy as a function of temperature.

Finally, the stability of the Au–Ag TdIh was estimated by computational calculations^60^ (Methodology section in the Supporting Information). The 9–18 configuration was tested (*q*-*v* Decmon notation).^16^ The model consisted of a Au_50_Ag_50_ alloyed core surrounded by a Au_15_Ag_85_ shell.^12^ The potential energy (*E*_pot_) is plotted versus temperature in Figure 5c. The TdIh structure was stable close to 1200 K, confirming its great stability (Movie S3). From the modeled structure, compositional line scans were acquired and compared with the experimental ones, having a good match between them and confirming the alloyed TdIh core–shell configuration and Ag segregation (Figure S13).

Nanostructures can be stable enough to be observed even if they are transitional structures^36,39^ or a product of coalescence to decrease the energy.^61,62^ Double icosahedra and anti-Mackay arrangements in bimetallic NPs have been described mainly theoretically for small clusters.^29,63−70^ Furthermore, the creation of secondary twins during MTP growth spurs symmetry breaking, triggering surface reconstruction and asymmetrical growth.^71−74^ For instance, Hendy and Doye^75^ discussed the presence of secondary twin planes and considered the formation of a twin plane at the outer layers of MTP and the hexagonal-compact packing (hcp)-terminating surface. This configuration stabilized the resulting structures.^75,76^ Anti-Mackay I-Dh clusters were described as a transitional structure in calculations for Au–Pt clusters^77^ and determined in ligand-protected Ag–core clusters and Au–Ag nanoclusters through X-ray diffraction analysis.^78−80^

Experimentally, larger structures were described by Rodríguez-López^18^ in bimetallic Dh-NPs with hcp surface reconstruction. It is well established that structural defects such as surface reconstruction,^16−18^ truncations,^16^ or vacancies^25^ are key factors in the growth kinetics driving the minimization of free energy and thus stabilizing them.^24^ Pelz et al.,^38^ on the basis of three-dimensional electron tomography, observed a twinned structure resulting from truncation during a Dh-to-Ih transition. The creation of secondary twins during MTP growth was discussed by El Koraychy and co-workers.^37^ They observed the initial covering of faulted islands on the facets of tetrahedral units, stabilized when new atomic layers nucleate on top of the faulted island, forming the TB that determines the shape evolution and stabilization of a Dh, Ih, or fragmented MTP. Xia and co-workers^81^ reported the transformation of octahedra to tetrahedra of Pt-NPs, observing the formation of two-dimensional islands at the edges of NPs forming fault defects that became the key symmetry-breaking mechanism for the autocatalytic transformation, supporting the key role of pure growth kinetics in structural transformations. Recently, El Koraychy and Ferrando^43^ reported the formation of exotic dIh and triple Ih in Cu@Au NPs from the evolution of a Dh seed. The structural features were modified depending on the seed type. Pure Cu-Dh seeds led to formation of a chiral Ih overlayer on all of the {111} facets and by subsequent surface reconstruction release of the strong in-plane compression, forming a dIh.

The TdIh structure could proceed from a similar pathway, forming faulted Ag-rich layers over an initial Au-rich I-Dh core fomented by the growth conditions. The stabilization of these secondary twin planes and further formation of partial tetrahedra could be enhanced by the crucial presence of Ag, because it acts as a directing agent for the development of twinned structures.^40,82,83^ These factors promoted the growth of the twinned shell, differing from the dIh by the double truncation originating Decmon-type surfaces in the absence of apical Dh tips.

In summary, we describe the novel structure of alloyed Au–Ag core–shell NPs. The NPs consisted of a Au–Ag core surrounded by a Ag-rich shell. HAADF imaging enabled the elucidation of their atomic arrangement, presenting an experimentally unreported structure, a doubly truncated double icosahedron, forming a twinned shell built by the formation of partial tetrahedral units over all of the facets of a central Ino-decahedral core. The experimental TdIh structure and growth pathway were assessed by HAADF simulations and computational calculations, highlighting the role of the alloy nature of the particles in their formation and stabilization. The observation of unconventional structures in bimetallic nanoparticles continues to highlight the complexity of nanoscale growth, which is highly dependent on the experimental conditions.

## Supporting Information Available

Experimental details, including additional STEM images, SAED, EDS analyses, focus and tilting series, synthesis variations, and image and structural simulations (PDF)
Back-and-forward focal series of a single TdIh NP (Movie S1) (AVI)
Growth pathway of an alloyed I-Dh to the TdIh (Movie S2) (AVI)
Heating simulation of the modeled TdIh, viewed in cross section (Movie S3) (AVI)

## Supplementary Material

nl3c04435_si_001.pdf

nl3c04435_si_002.avi

nl3c04435_si_003.avi

nl3c04435_si_004.avi
