# Supplementary material for: Experimental High-Resolution Observation of the Truncated Double-Icosahedron Structure: A Stable Twinned Shell in Alloyed Au–Ag Core@Shell Nanoparticles
Source: Nano Lett. 2024 Apr 1;24(14):4072–81. doi: 10.1021/acs.nanolett.3c04435 (PMC11010228; doi:10.1021/acs.nanolett.3c04435)
Supplement: Supplementary file 1 — nl3c04435_si_001.pdf [file nl3c04435_si_001.pdf]

Supporting Information

Experimental High-Resolution Observation of the Truncated Double Icosahedron Structure: A Stable Twinned-Shell in Alloyed Au-Ag Core@Shell Nanoparticles

Rubén Mendoza-Cruz;[ξ]* Juan Pedro Palomares-Báez;[γ] Stephan Mario López-López;[ξ,§] Juan Martín Montejano-Carrizales;[φ] José Luis Rodríguez López; [ə] Miguel José Yacamán;[ɋ] and Lourdes Bazán-Díaz. [ξ]

[ξ] *Instituto de Investigaciones en Materiales, Universidad Nacional Autónoma de México. Circuito Exterior, Ciudad Universitaria, Ciudad de México, México, 04510.*

[γ] *Facultad de Ciencias Químicas, Universidad Autónoma de Chihuahua. Circuito Universitario s/n, Campus II, Chihuahua 31125, Chih., México.*

[§] *Posgrado en Ciencia e Ingeniería de Materiales, Universidad Nacional Autónoma de México. Circuito Exterior, Ciudad Universitaria, Ciudad de México, México, 04510.*

[φ] *Instituto de Física, Universidad Autónoma de San Luis Potosí. San Luis Potosí, México, 78290.*

[ə] *Advanced Materials Department, Instituto Potosino de Investigación Científica y Tecnológica, A.C., 78216 San Luis Potosí, México.*

[ɋ]*Department of Applied Physics and Materials Science, and MIRA, Northern Arizona University. Flagstaff, Arizona, USA, 86011.*

**Correspondence to:*

e-mail: rmendoza@materiales.unam.mx

Methodology

a) *Reagents.* Gold chloride, AuCl (Sigma-Aldrich, 99.9%), silver nitrate, $AgNO_3$ (Sigma-Aldrich, >99%), oleylamine (OLA, Sigma-Aldrich, 70%), and oleic acid (OAc), were purchased and used without further treatments.

b) *Synthesis of Au-Ag NPs*. The synthesis was adapted from the one described by Guisbers et al.[1] Gold and silver salts were reduced simultaneously using OLA and OAc as reducers, stabilizers, and solvents. Briefly, 7 mM of gold chloride and 21 mM of silver nitrate were mixed in a 5 ml solution of 1:10 oleylamine/oleic acid at room temperature and heated for 90 minutes at 120°C. Then, the mixture was heated at 250°C for 120 minutes and cooled to room temperature. Finally, the solution was washed with ethanol three times by centrifugation (3100 *g*) and dispersed with chloroform. To gain information on the growth process of the particles, additional experiments were conducted, one at initial temperature of 150°C, and the second one in the absence of OA. Aliquots were taken at early stages of reaction to observe the formed particles at this reaction stage.

c) *Structural characterization*. The structure of the prepared Au-Ag NPs was determined through transmission electron microscopy using a JEOL ARM200F microscope operated at 200 keV, equipped with a CESCOR spherical aberration (Cs) corrector and an Oxford AZtecTEM detector for energy-dispersive X-ray spectroscopy (EDS), (RRID:SCR_024400). Images were captured in high-angle annular dark-field (HAADF) mode with convergence and collection semi-angles of 24 mrad and 54-220 mrad, respectively. EDS mapping and line-scans were acquired to determine the NPs chemical composition. The composition of the core and shell was estimated from the compositional maps by reconstructing the corresponding spectra of the specific areas at the core and shell, and considering a core and shell sizes of 6.4 nm and 1.6 nm, respectively. Scanning transmission electron microscopy (STEM) images were simulated to compare with the experimental ones using the QStem software developed by C. Koch,[2] based on the experimental conditions for data collection.

d) Molecular Dynamics and Molecular-Dynamics/Monte Carlo simulations: All computational calculations were conducted using the LAMMPS software[3] with a MEAM (Modified Embedded Atom Method) type interaction potential for atom interactions.[4] The 9-13 configuration was tested according to the *q-v* notation of the Decmon structure.[5] Starting from a perfect core-shell structure ($Au_{25}@Ag_{75}$), a hybrid MD/MC (Molecular Dynamics/Monte Carlo) simulation was carried out in which every 100 Molecular Dynamics steps, 10 atomic swaps between Au and

Ag atoms were attempted. The simulation was carried out at a temperature of 100 K in an NVT ensemble, using a Noose-Hover type thermostat. During the simulation, at a certain number of steps snapshots of the particle (*xyz* coordinates) were saved, among which the one in which the experimentally obtained proportions were obtained (with core with proportions 50-50 and shell with proportions 20-80) was chosen. Once the particle with the appropriate proportions was obtained, the atomic positions were optimized and line scans (histograms) were performed in the *X* direction. The line scans were performed after applying random rotations in the three axes. For the heating simulations, a temperature ramp from 1 to 1400 K was applied with a heating rate of 0.1 K/ps, using the same thermostat as before.

Supplementary Figures:

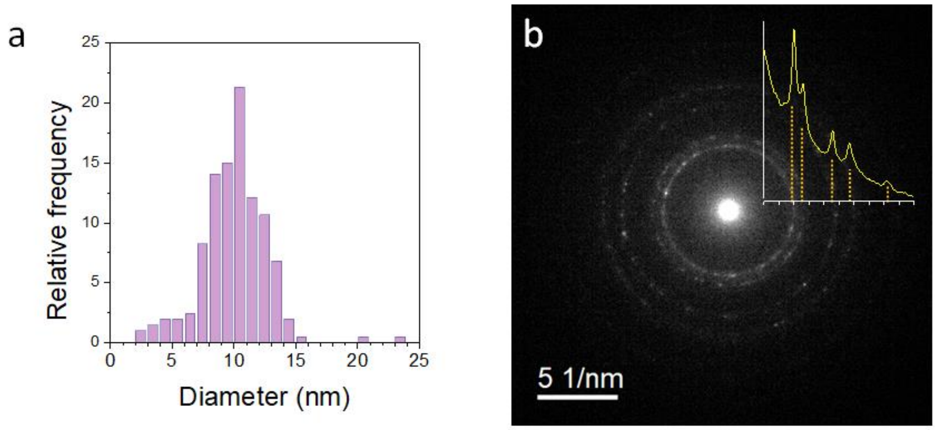

Figure S1. (a) Size histrogram of the synthesized Au@Ag NPs. The calculated average size was 10.1 ± 2.6 nm. (b) Selected-area electron diffraction of a small group of NPs.

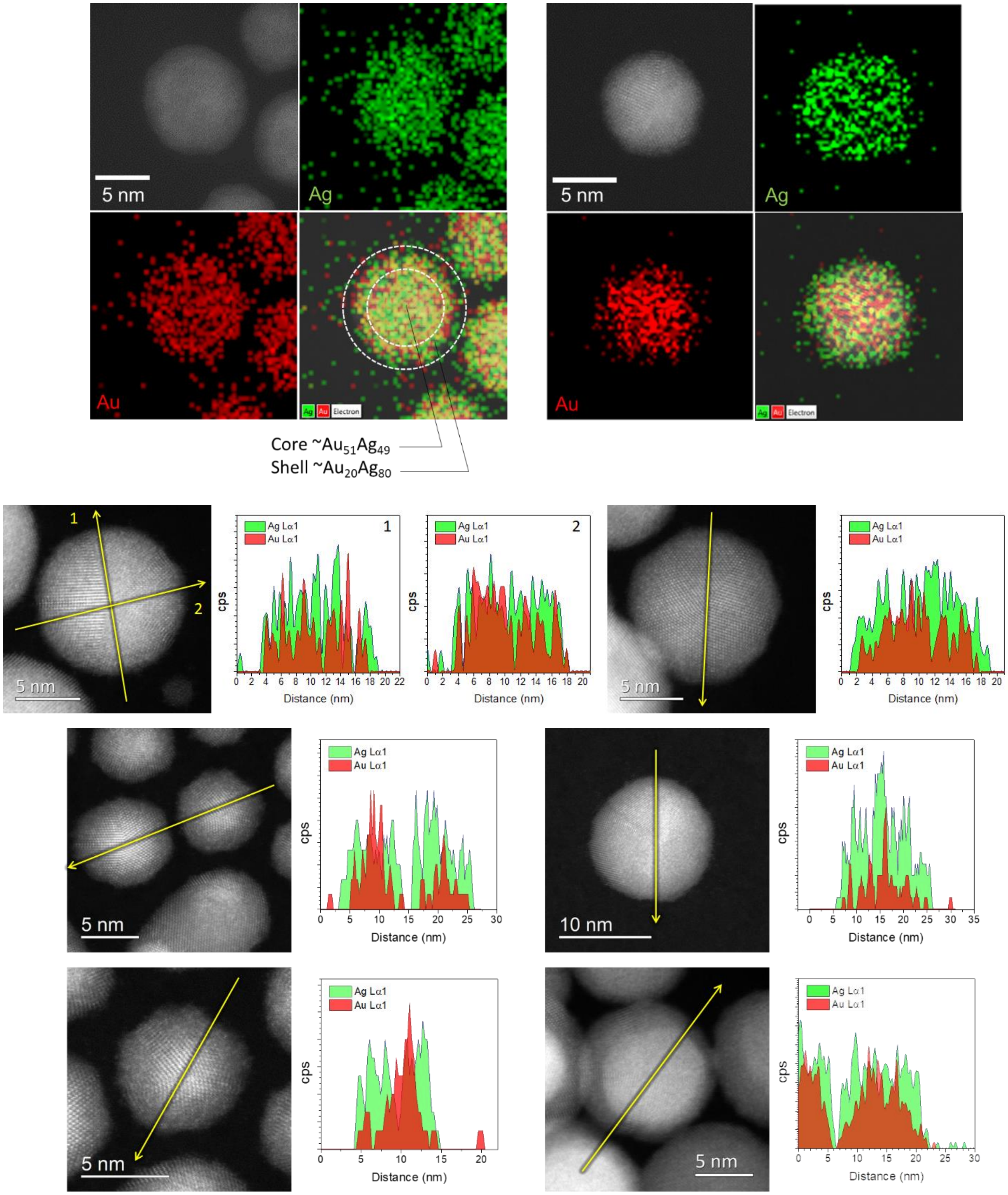

Figure S2. EDS mappings (up) and EDS linescans (bottom) across single Au@Ag NPs. Au and Ag are present into the particle structure, forming a core-shell type structure with an Ag-rich shell. The estimated core and shell compositions were $Au_{51}Ag_{49}$ and $Au_{20}Ag_{80}$, respectively, and Ag segregation to the surface is observed.

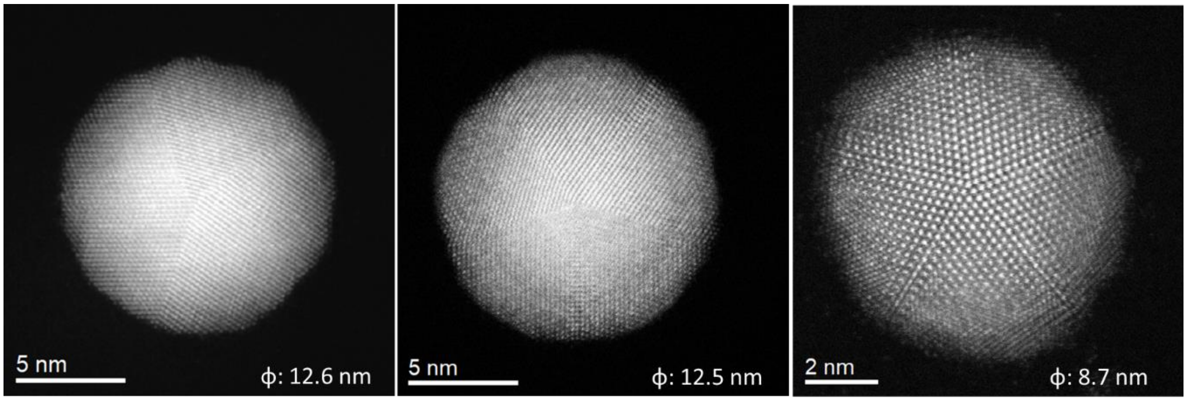

Figure S3. HAADF-STEM images of a regular gold Dh (left) and a herein reported Au-Ag NPs (middle and right) with different diameters. The particles were imaged near to the main five-fold axis. Clear differences are observed.

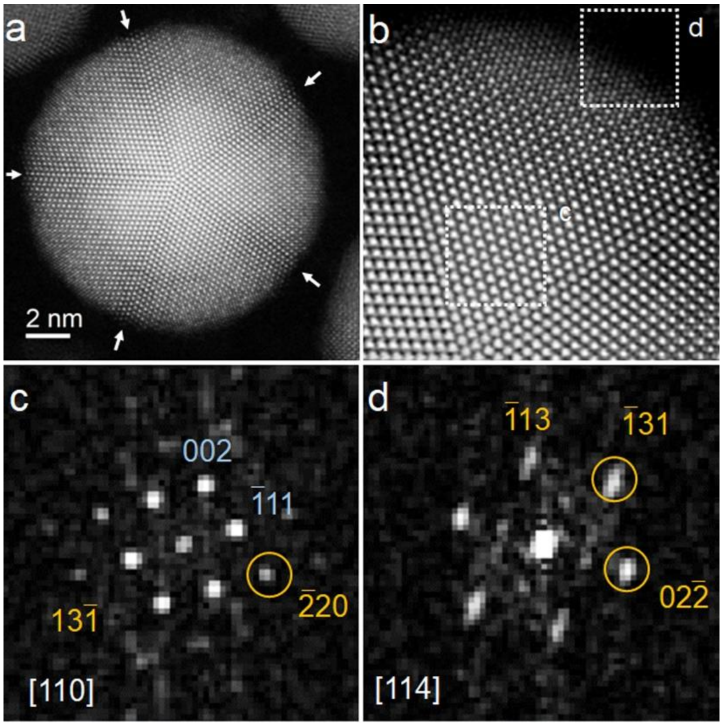

Figure S4. HAADF-STEM images and FFT analysis of the synthesized Au@Ag NPs.

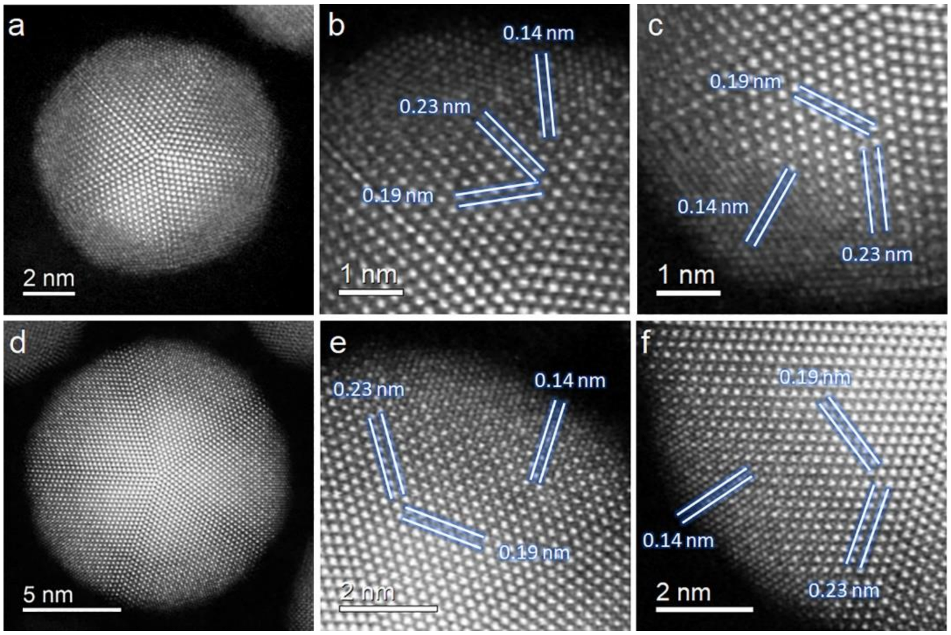

Figure S5. HAADF-STEM images and lattice distances measurements of the synthesized Au@Ag NPs.

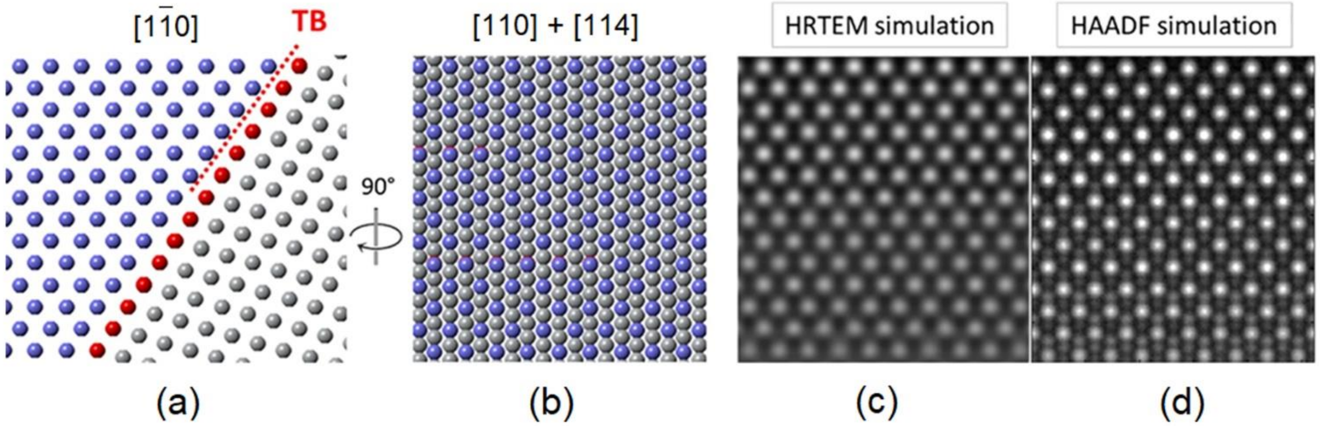

Figure S6. Atomistic models of a twinned crystal viewed along the (a) [1$\bar{1}$0] and (b) [110] + [114] directions. (c) Conventional HRTEM and (d) aberration-corrected HAADF simulated images of the [110] + [114] twinned crystal.

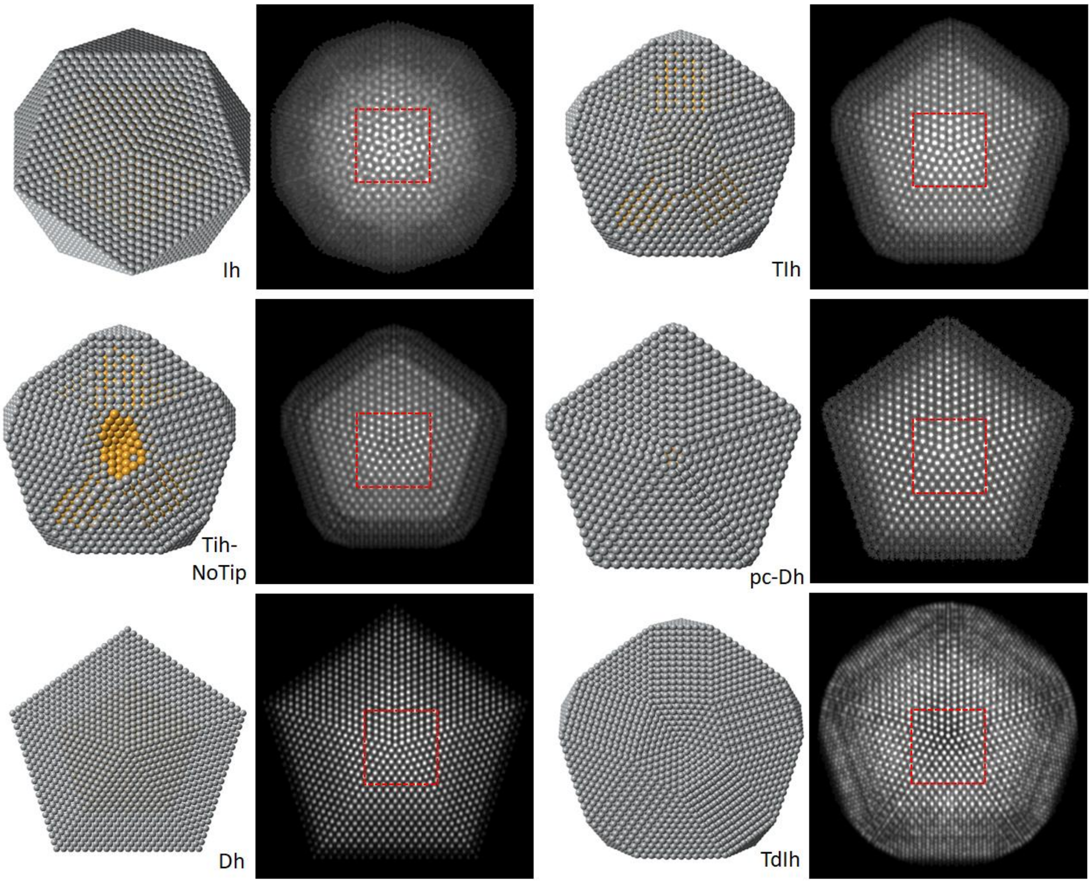

Figure S7. Atomistic models and HAADF simulations for the icosahedron (Ih), single-truncated icosahedron (TIh), single-truncated icosahedron with no Dh-tip (TIh-NoTip), partially-covered decahedron (pc-Dh), regular decahedron (Dh), and the doubly-truncated double icosahedron (TdIh), viewed from the five-fold axis. The central area in enclosed to highlight the different contrast patterns.

When truncation occurred to form a TIh, the particular characteristics at the NP center (at the vicinity of the five-fold axis) slightly remained, while the change in the contrast across the twin boundaries indicated the rising of {100} facets. When a pc-Dh is formed, the NP center contrast resembles the regular Dh because of the practically absence of the different way of truncation of the Dh-tip, while an asymmetric contrast along the TB is observed.

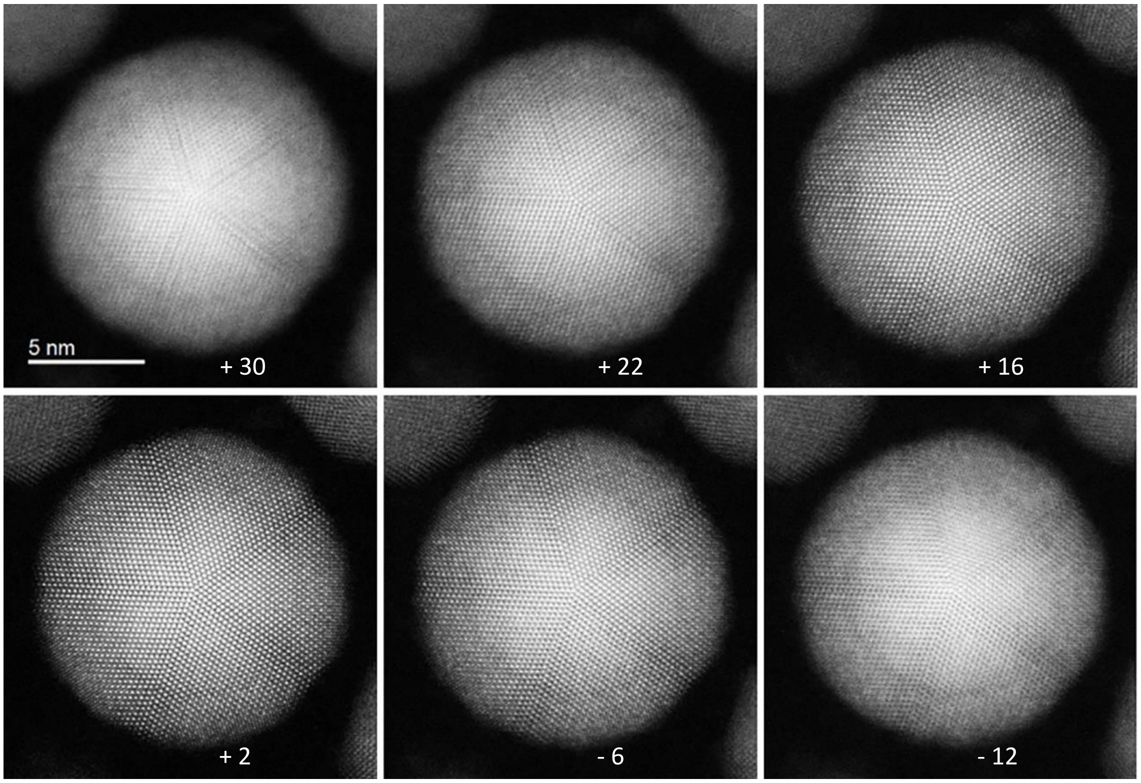

Figure S8. Focal series of a single Au-Ag NP oriented along the main five-fold symmetry axis. The images were acquired at +30 nm, +22 nm, +16 nm, +2 nm, -6 nm y -12 nm focus condition. When imaging an NP at an overfocus condition, strong features at the TB appeared, suggesting the presence of {100} facets. When focusing downwards, atomic [110] contrast appeared across the particle. At this five-fold symmetry orientation, the contrast is dominated by the [110]-oriented parent Dh core. When under focus continues, the contrast from the twinned shell becomes evident as its thickness increases at the edges. This thickness and the compositional variations between the core and shell are responsible of the apparent larger size of atomic columns at the NP center. At the final under focus condition, again, the features at the TB became more visible with slight differences, suggesting a non-symmetrical shell on the top and bottom for this NP. It is worth noting that during the focal series, the structure of the particle remained unaltered, providing insight into the stability of the reported structure. Particle drift was present extending the focus condition.

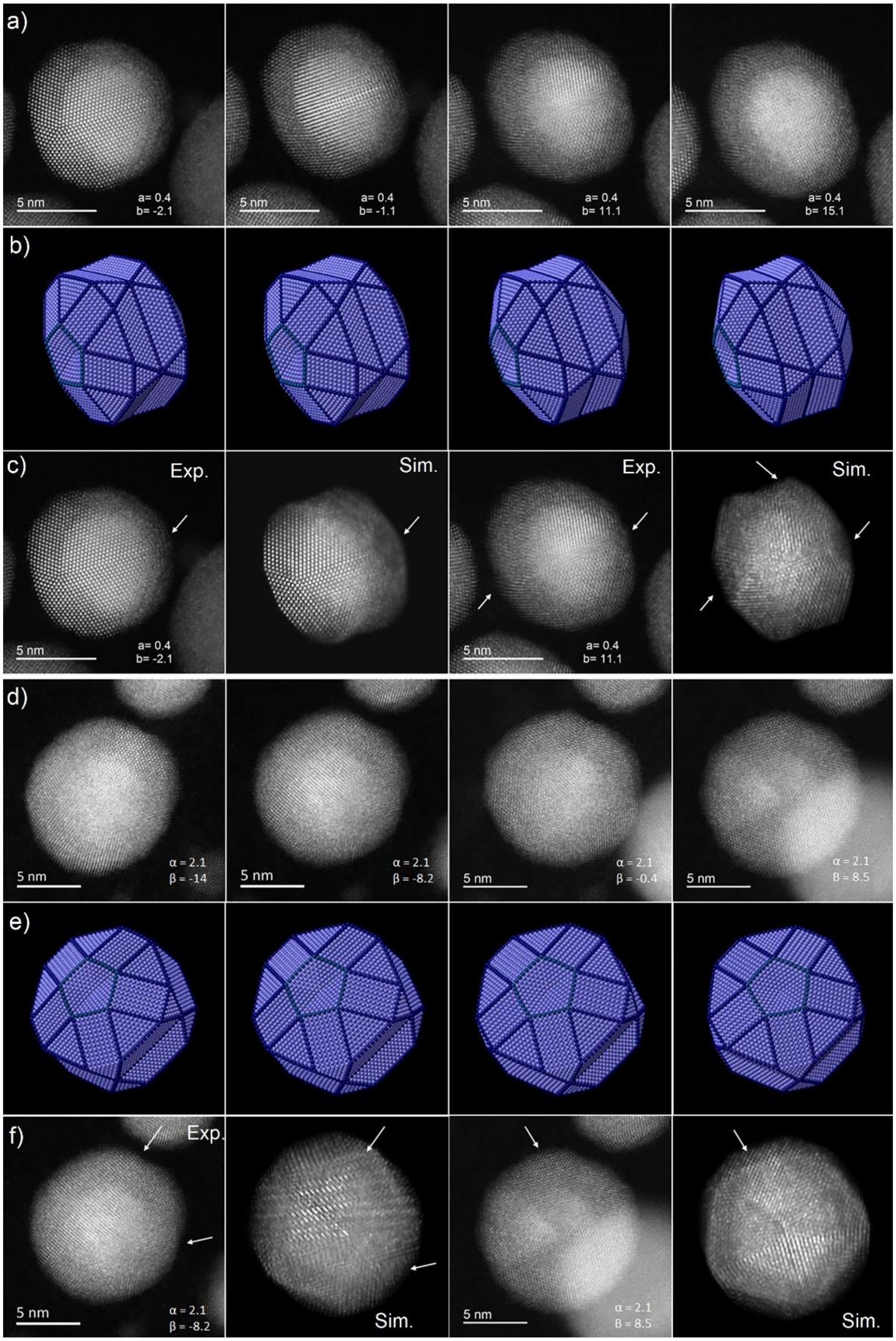

Figure S9. (a,d) HAADF-STEM tilting series of two single Au-Ag nanoparticles. The sample was tilted around the β angle. (b,e) Atomistic models of the imaged particles. (c,f) Experimental and simulated HAADF-STEM images. The arrows highlight the great match with between specific characteristics of the NPs in both images.

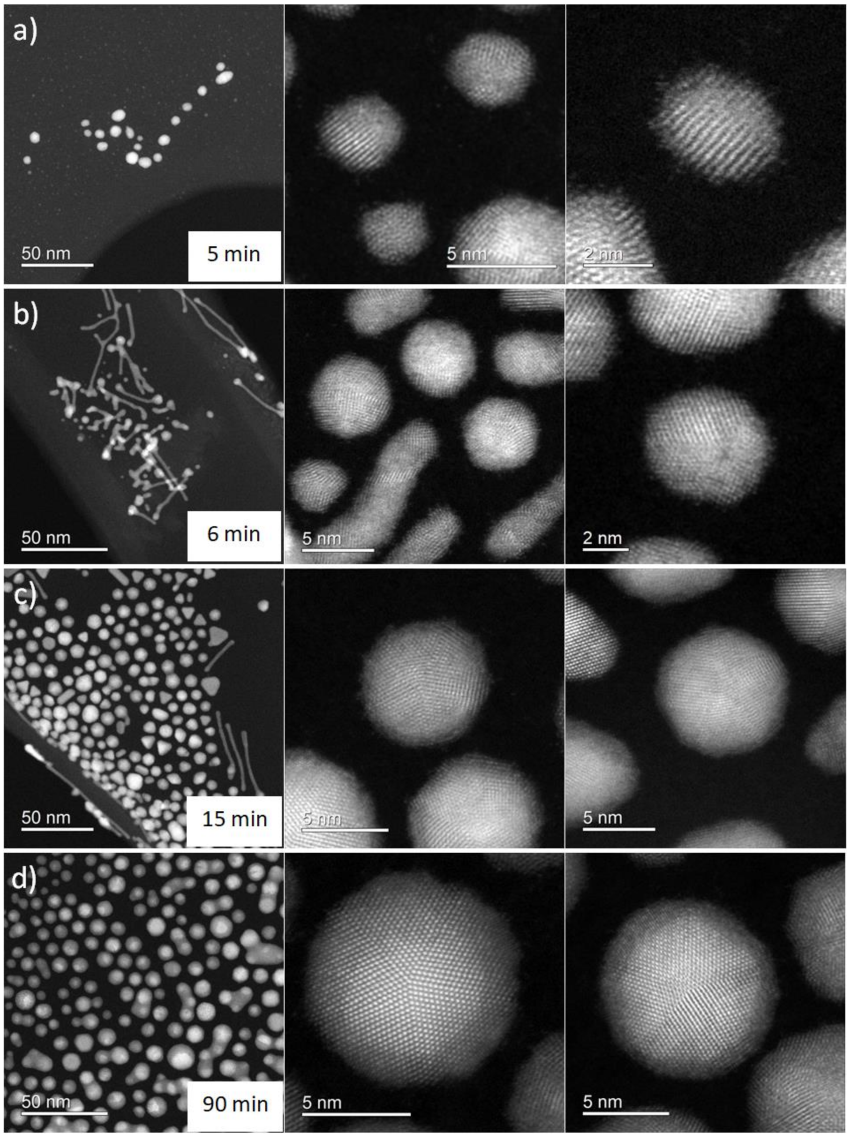

e)

Time (min)	Au %at	Ag %at	Size (nm)
**5**	71	29	3.6
**6**	70	30	4.5
**15**	61	39	7.1
**90**	27	73	8.3

Figure S10. Growth evolution experiments taking aliquots at different reaction times. (a) At 5 minutes the initial particles corresponded to small I-Dh with an average composition of Au71-Ag29. (b) Then, silver concentration increased with time, starting reconstruction after 6 minutes of reaction. Some low-temperature ultrathin nanowires formation is observed. (c) After 15 minutes, the TdIh structure is visible, with so far small shells. (d) After 90 minutes the particles showed the TdIh structure with the overall Ag-rich composition (Au27-Ag73). (e) Table showing the evolution of size and chemical composition determined by EDS as a function of reaction time.

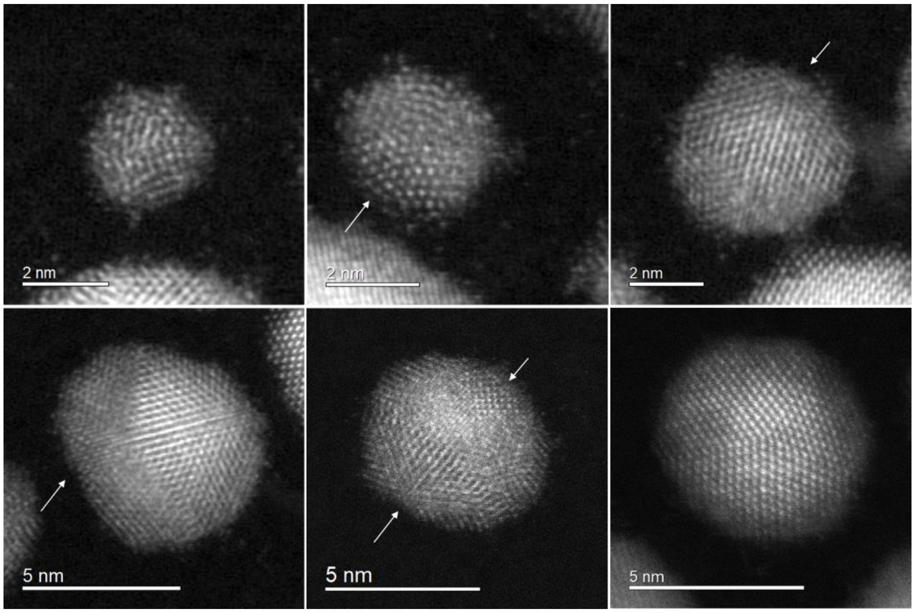

Figure S11. HAADF-STEM images of small cores of different size (from smaller to larger) at early growth stages. The arrows point at features that indicate the reconstructed structure of these particles, i.e. these features are present in the truncated double icosahedral structure.

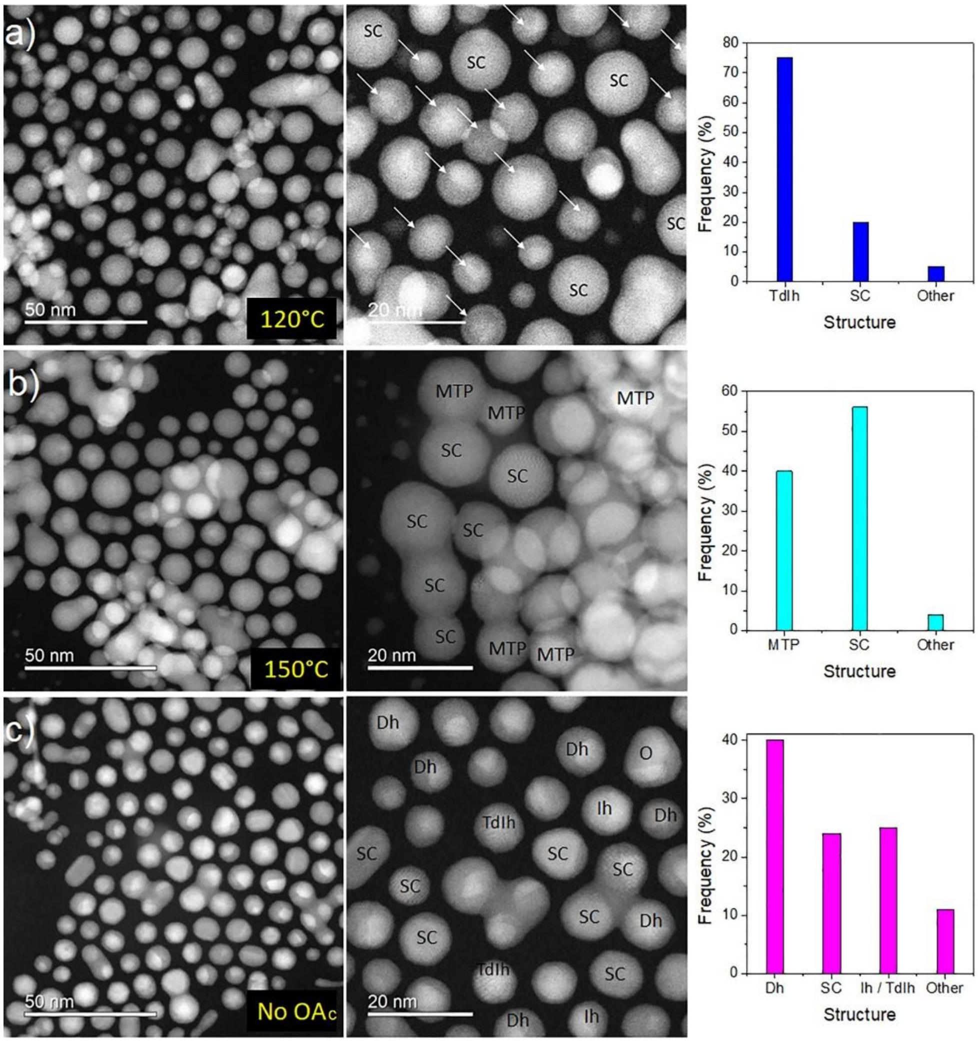

Figure S12. HAADF-STEM images and structural yield of the obtained NPs when the synthesis was performed at a) 120°C, b) 150°, and c) the absence of OAc. At higher temperature (150°C) the formation of single-crystal NPs was favored, yielding less MTP structures. In the absence of OA, Dh MTP are favored, followed by single crystalline particles and Ih/TdIh structures. Other particles involved single-twin structures or plate-like shapes. TdIh presence could have been unaware in previous reports due to the similarity in the projected structure from TEM analysis.

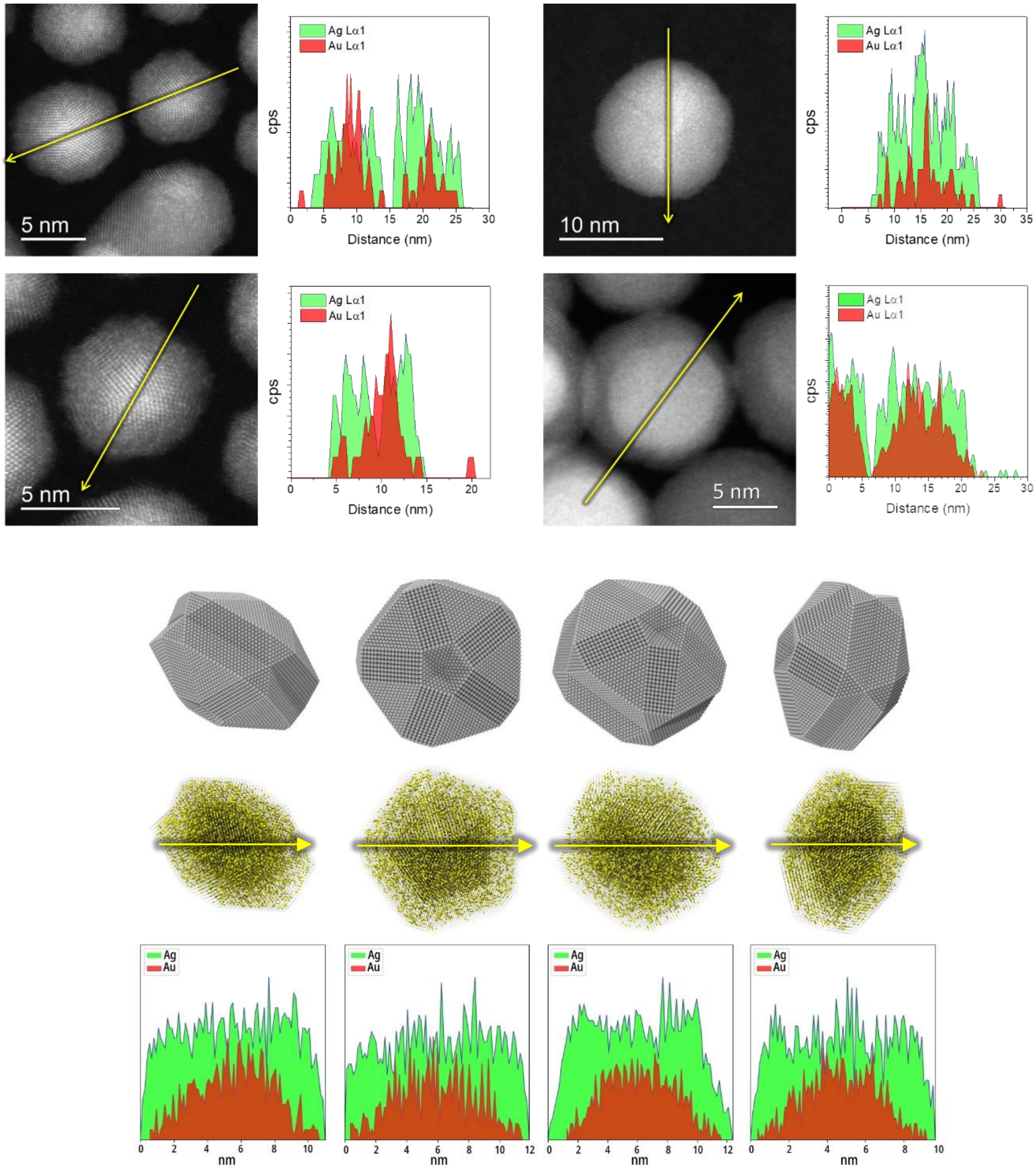

Figure S13. Experimental and calculated compositional linescans of the TdIh structure. The modeled TdIh was obtained using a hybrid MD/MC method along different directions. As observed, the particle present an alloyed Au-Ag core surrounded by an alloyed Ag-rich shell, with Ag-segregated at the outer layers. The calculated linescans had a good match with the experimental EDS spectra (upper Figures and **Figure S2**).

Supplementary Videos:

Supplementary videoclips were included.

Videoclip 1: Back-and-forward Focal series on a single TdIh NP.

Videoclip 2: Growth pathway of an alloyed I-Dh to the TdIh.

Videoclip 3: Heating simulation of the modeled TdIh, viewed in cross-section.

References

1. Guisbiers, G.; Mendoza-Cruz, R.; Bazan-Diaz, L.; Velazquez-Salazar, J. J.; Mendoza-Perez, R.; Robledo-Torres, J. A.; Rodriguez-Lopez, J. L.; Montejano-Carrizales, J. M.; Whetten, R. L.; Jose-Yacaman, M., Electrum, the Gold-Silver Alloy, from the Bulk Scale to the Nanoscale: Synthesis, Properties, and Segregation Rules. *ACS Nano* **2016,** *10* (1), 188-198.
2. Koch, C. T., *Determination of core structure periodicity and point defect density along dislocations*. Arizona State University: 2002.
3. Thompson, A. P.; Aktulga, H. M.; Berger, R.; Bolintineanu, D. S.; Brown, W. M.; Crozier, P. S.; in 't Veld, P. J.; Kohlmeyer, A.; Moore, S. G.; Nguyen, T. D.; Shan, R.; Stevens, M. J.; Tranchida, J.; Trott, C.; Plimpton, S. J., LAMMPS - a flexible simulation tool for particle-based materials modeling at the atomic, meso, and continuum scales. *Computer Physics Communications* **2022,** *271*, 108171.
4. Alvi, S. M. A. A.; Faiyad, A.; Munshi, M. A. M.; Motalab, M.; Islam, M. M.; Saha, S., Cyclic and tensile deformations of Gold–Silver core shell systems using newly parameterized MEAM potential. *Mechanics of Materials* **2022,** *169*, 104304.
5. Palomares-Baez, J. P.; Montejano-Carrizales, J. M.; Guisbiers, G.; Jose-Yacaman, M.; Rodriguez-Lopez, J. L., The Decmon: a new nanoparticle shape along the truncation path from the icosahedron to the decahedron. *Nanotechnology* **2019,** *30* (42).
